# How Do People Experiencing Schizophrenia Spectrum Disorders or Other Psychotic Disorders Use the Internet to Get Information on Their Mental Health? Literature Review and Recommendations

**DOI:** 10.2196/mental.5946

**Published:** 2017-01-03

**Authors:** Murielle Villani, Viviane Kovess-Masfety

**Affiliations:** ^1^ Fondation Pierre Deniker Paris France; ^2^ Laboratoire de Psychopathologie et Processus de Santé Université Paris Descartes Boulogne-Billancourt France; ^3^ École des Hautes Études en Santé Publique Paris France

**Keywords:** Internet, health information, e-mental health, e-support, schizophrenia spectrum disorders, psychotic disorders

## Abstract

**Background:**

Studies show that the Internet has become an influential source of information for people experiencing serious psychiatric conditions such as schizophrenia spectrum disorders or other psychotic disorders, among which the rate of Internet users is growing, with rates ranging from 33.3% to 79.5% given the country. Between 20.5% and 56.4% of these Internet users seek mental health information.

**Objective:**

Focusing on this population’s Web searches about their mental health, this paper examines what type of content they look for and what could be the benefits and disadvantages of this navigation.

**Methods:**

We conducted a literature review through medical and psychological databases between 2000 and 2015 using the keywords “Internet,” “Web,” “virtual,” “health information,” “schizophrenia,” “psychosis,” “e-mental health,” “e-support,” and “telepsychiatry.”

**Results:**

People experiencing schizophrenia spectrum disorders or other psychotic disorders wish to find on the Internet trustful, nonstigmatizing information about their disease, flexibility, security standards, and positive peer-to-peer exchanges. E-mental health also appears to be desired by a substantial proportion of them. In this field, the current developments towards intervention and early prevention in the areas of depression and bipolar and anxiety disorders become more and more operational for schizophrenia spectrum disorders and other psychotic disorders as well. The many benefits of the Internet as a source of information and support, such as empowerment, enhancement of self-esteem, relief from peer information, better social interactions, and more available care, seem to outbalance the difficulties.

**Conclusions:**

In this paper, after discussing the challenges related to the various aspects of the emergence of the Internet into the life of people experiencing schizophrenia spectrum disorders or other psychotic disorders, we will suggest areas of future research and practical recommendations for this major transition.

## Introduction

Health information–seeking is the most common online activity in today’s world [1], and 50 million websites are devoted to health-related information [2]. Internet, social media, and online technologies have become powerful tools in the mental health sector, leading transformations that go from infodemiology and infoveillance—the monitoring of suicidal tendancies [3-6] or medical antidepressants prescription behaviors [7], for example, with a Google Trends-based approach—to crowdsourcing for conducting randomized trials for the purpose of scientific research [8], not to mention the delivery of evidence-based therapies targeting various mental illnesses [9,10]. Some thought papers have studied the evolution of telepsychiatry, also called “e-mental health care,” where this expression defines a “practice supported by electronic processes and communication, (including) mental health applications and links on mobile phones” and services of “information (delivering), peer support services, computer- and Internet-based programs, virtual applications, and games, as well as real time interaction with trained clinicians” [11]. Thus, a large variety of interventions are designated alternatively by the terms eHealth and e-support or mHealth and m-support when online technologies are reached via mobile or smartphone. Research has stressed the fact that this emerging kind of support is a cost-effective and ongoing resource that defies geographical, timely, and social barriers (the “mental health gap”) [12,13] without replacing the traditional system [14,15]. While there has been an increase in the number of people using the Internet for information and support relating to mental health disorders, available resources—from psychoeducation to self-help therapy, peer-to-peer support forums, and e-counseling—focus mainly on depression, anxiety, and bipolar disorder, with a particular interest in the monitoring of signs and symptoms by smartphone or digital application [10], together with psychoeducational programs [9] and not so much on schizophrenia spectrum disorders or other psychotic disorders.

People facing severe psychiatric conditions have become more and more active in their treatment and care plan, which is partly due to the legal evolutions of the last decades but also an increasing empowerment of people experiencing mental illnesses and their representing associations [16,17]. Getting information on their disease by themselves contributes to this new balance in the relationship with their doctor. Finally, the change of paradigm about schizophrenia spectrum disorders or other psychotic disorders in the scientific community, with the emergence of the possibility of recovery [18], giving hope to people experiencing those mental illnesses, has also encouraged them to actively seek help, support, and reassurance by using different resources. The Internet is one of those resources and offers unprecedented access to information shared by professionals and peers.

People experiencing stigma, who avoid face-to-face contacts as much as possible, are more likely to use the Internet for accessing health information than those with conditions that do not come with negative stereotypes [19]. People with schizophrenia, a disease that is particularly subject to stigma [20-23], should be well represented among Internet users. As a general background, the relatively scarce but quickly growing body of research on the use of the Internet for mental health–related information among people experiencing serious psychiatric conditions shows that in developed countries where there are available studies on this matter, more and more people living with such conditions use the Internet and a relatively substantial proportion does so in order to access mental health information. Indeed, in populations specifically constituted of people experiencing severe psychiatric conditions, the percentage of Internet users ranged from 33.3% in 2009 the United States [24] to 62.6% in 2008 in Switzerland [25] and 79.5% in 2014 in Germany [26], with the highest rates corresponding to the latest studies. The percentage of people with a serious mental illness or psychiatric condition seeking health-related information on the Internet was 42.9% in Switzerland [25], 53% in the United States [24], and 56.36% in Germany [26]. A recent American study about the connectivity of people with schizophrenia investigated the daily use of social media network websites and found that 27% of the participants used these sites everyday [27]. The predictive factors of this usage are various, including young age and higher education [24], knowledge of English, and private Internet access [25]. As one can also access the Internet through smartphones, the prevalence of smartphone usage among people experiencing schizophrenia is also of interest. In this area, studies have found that a large majority of participants had a cell phone, knew how to text, and were able to use cell phones with few problems [27-29]. The link with specific diagnoses and their severity is controversial: while some studies highlight that people experiencing schizophrenia have a lower access to the Internet due to financial problems, an itinerant way of life, or a low education level [1], more recent research studies have found that the actual Internet usage is not different for these persons [26,30].

In this evolving context, it seems important to further explore the use of the Internet among people experiencing schizophrenia or other psychotic disorders in relationship to their mental health. Our specific research questions include the content of the information looked for and retrieved; the benefits in terms of empowerment, stigma resistance, coping, social interactions, management of the care process, and clinical improvement; and the difficulties they might encounter (for example, the potential risk of aggravating symptoms) in comparison with the absence of use of the Internet. We then suggest recommendations about practical evolutions in this promising field.

## Methods

The aim of our review is to identify published literature that would help assess the Internet and Internet-based technology use by people experiencing schizophrenia spectrum disorders or other psychotic disorders, in particular regarding information about their health. The scope of our research included people experiencing schizophrenia spectrum disorders as well as other psychotic disorders [31]. However, we have included in our review some studies investigating people experiencing schizophrenia spectrum disorders or other psychotic disorders’ Internet use while sometimes using other terminologies, such as “psychosis,” “severe or serious mental illness,” or “serious psychiatric conditions or disorders.” We focused on definitions of Internet use that did not specifically addressed addiction issues or the use of a virtual environment as a therapy in itself.

The analysis of people experiencing schizophrenia spectrum disorders or other psychotic disorders’ Internet use being relatively recent, most of the studies in that field include small populations and heterogeneous methodologies, which presents an increased risk of error in the context of a meta-analysis [21]. Consequently, we have done as much as we could to approximate a systematic review by following the Preferred Reporting Items for Systematic Reviews and Meta-Analyses guidelines; indeed, our motivation was not to measure the effectiveness of Internet use for our target population but to discuss specific aspects of the evolution in this matter in both a contextual and a theoretical approach.

Therefore, we conducted a literature review through the medical and psychological databases PsycInfo, PsycArticles, SantéPsy, Cairn, Medline, Wiley Interscience, and PubPsych between 2000 and 2015 (including those complete years) using the keywords “Internet,” “Web,” “virtual,” “health information,” “schizophrenia,” and “psychosis.” Articles were included only if their scope addressed mainly or subsequently a population with schizophrenia spectrum and other psychotic disorders and if their main topic was related to their Internet use or Internet-based technologies use. Pertinent articles retrieved allowed us to broaden our search, for example, by using additional keywords (“eMental health,” “eSupport,” “telepsychiatry,” “mobile,” “online”). Subsequently, all additional relevant articles cited in reference lists were added to our sources of information.

Out of 85 papers retrieved, 11 were removed as outside of our scope and 45 articles were identified as pertinent for our research, read in full text, and used for this paper with their main qualitative and quantitative findings recorded. The body of articles analyzed allowed us to identify themes and issues that are described in our results. Each included article was then re-reviewed in the light of the themes that emerged from our first analysis. The remaining 29 more general papers were kept in order to feed the discussion and are quoted in the paper as well.

## Results

### What Are People Experiencing Schizophrenia Spectrum Disorders or Other Psychotic Disorders Looking for on the Internet?

#### Type of Information Requested

In most of the cases, people experiencing schizophrenia spectrum disorders or other psychotic disorders who are regular or occasional Internet users seek health-related information [15,25,26]. With an average contact with their doctor lasting about 15 minutes every 6 months, one can understand that people experiencing schizophrenia may be tempted to seek information on their own [2]. Kalckreuth et al [26] have ranked the different kinds of health information for which people experiencing psychiatric conditions look. General information about mental disorders such as explanations about diagnosis, symptoms, and prognosis comes first (32%), followed by general information on medication such as description of secondary effects (25%). Connected people experiencing severe mental illness also look for information about mental health services: where and how to access them, what quality to expect (22%), and platforms of exchange with peers (11%) or professionals (10%) [26].

A qualitative Austrian study investigating the nature and consequences of health-related Internet use among people experiencing schizophrenia spectrum disorders [32] reported that the Internet is an important and influential source of information for them and that their behavior on the Internet is not significantly different from nonaffected people’s behavior. Their subjects of interest on the Internet are general information about schizophrenia, treatments and secondary effects (especially about how to avoid them), diagnosis verification, services access and evaluation, risk factors, and causes [32].

#### Preferences About Internet Site Characteristics

The Austrian study, analyzing 26 individual semistructured interviews, identified precise elements concerning the content and quality that people experiencing schizophrenia spectrum disorders or other psychotic disorders are searching for on the Web while accessing information about their mental health problem. The quality and reliability of Internet information is judged overall satisfactory by them, with a preference for official pages from universities and magazines over private pages or chat rooms. The evaluation by the users is a function of clarity; usefulness; transparency; advice of family, friends, and relatives; and the link with the users’ personal experience. It appears that people experiencing schizophrenia spectrum disorders or other psychotic disorders mainly seek clear, objective, scientific, and actively “destigmatizing” information. They value the points of view and positive case stories of people experiencing the same mental illness, further education about drugs, more doctor recommendations of good websites, and more opportunities to discuss with their doctor about what is found on the Internet [32].

#### Use of Peer-to-Peer Exchanges

Among Internet users experiencing schizophrenia spectrum disorders or other psychotic disorders, those with a higher frequency of access also used it as social media [26] or a possibility of disclosure [15]. Research analyzing 1200 postings of 576 users in 12 international schizophrenia Internet forums [33] established that, within this kind of usage, people experiencing schizophrenia spectrum disorders or other psychotic disorders’ goals are to find and exchange information mainly about diagnosis and daily problems, such as medication, with peers. Interestingly, on the relatively small number of existing schizophrenia forums, one can find that affected users have the same behavior as any other user. Their preferred self-help mechanisms are disclosure and providing information, followed by sharing emotions. The main pursued goal is to exchange information. Other valuable characteristics of Internet forums for them are anonymity and a limited level of commitment [33]. As far as peer-to-peer exchanges are concerned, people experiencing schizophrenia spectrum disorders or other psychotic disorders who are already users have expressed wishes for structured, moderated, and secure support forums [32].

#### Use of e-Support and m-Support

Taking place in a digital and virtual environment, Internet interventions designed for people experiencing schizophrenia spectrum disorders or other psychotic disorders have no delivery costs and allow dissemination of specialized interventions without geographic barriers. They can reduce perceived stigma by eliminating face-to-face contacts, which can be particularly appreciated in the context of this condition. Last, they can provide ongoing resources that are available at any time of day and night [14,34,35]. The Internet is also well adapted to the challenges of early intervention, as it appears particularly useful for young people. In addition, the disinhibiting effect of online communications and the suppression of face-to-face contact present interesting opportunities for those who are socially impaired [32].

Service users have expressed interest in those digital interventions that complement or supplement real world initiatives. The German study cited earlier established that among Internet users experiencing psychiatric conditions, 27.6% wished to take part in Web-based programs for mental health self-management [26]. Other research studies have shown that a majority of participants expressed an interest in text message medication reminders after hospital discharge [29] and that young people and other tech-friendly people experiencing psychosis generally ask for more connectivity in treatment [36,37].

Consequently, Internet-based interventions for schizophrenia spectrum disorders or other psychotic disorders have been studied as pilot protocols and are becoming more and more operational [14,34,35]:

- Online peer support forums, which have showed robust links with empowerment and recovery, if structured and moderated (otherwise, the level of distress can be raised) [14] or online peer-based support programs [35,38]
- Online patient psychoeducation [39], which is a cost-effective and acceptable method preferred by younger people [36] and those familiar with new technologies
- Web-based cognitive behavioral therapies using interactive exercises and multimedia materials (eg, video, audio) and targeting specific problems such as auditory hallucinations [40], social cognition [41], or depression on mood and positive symptoms [42]
- Virtual reality programs targeting 3 dimensions of social behavior: facial emotion recognition, social anxiety, and conversation time [43]
- Tablet computers using multimedia tools to promote personal recovery [44]
- Mobile-based interventions, which offer, with easy-to-use phones, great promise for transforming treatment delivery in addressing psychosis and the opportunity to deliver evidence-based tailored interventions; enable real-time support; gather ecologically valid information; efficiently control signs of relapse [45]; and improve medication adherence, auditory hallucination prevalence, and social interactions [46]
- Online family interventions, which improve accessibility and knowledge for family
- Early intervention, which can be revolutionized by new technologies with the access to and the engagement with specialized early intervention services offering an extended support and a comprehensive risk management framework [14,37]

#### Examples of Existing Technologies

##### Personal Control in Rehabilitation

This is an Internet platform for people experiencing schizophrenia spectrum disorders or other psychotic disorders and their caregivers in the Netherlands that has provided satisfaction to its users and proved to be helpful in reaching more self-management by service users; facilitating communication between them and their caregivers; and matching treatment, support, and relapse prevention [47]. The service users have been able to communicate, make appointments, give feedback, and look for information about the disease and its treatment. The tool has enabled them to gain better accessibility to caregivers, maintain contacts with peers experiencing the same mental illness, and describe experiences. They have felt relief in being able to write down worries and questions. They have expressed the need for more focus on rehabilitation and less on disease and treatment, more attention on improving personal skills, more interactivity, and more possibilities to control who could look into their personal data. The use was not informal; both people experiencing schizophrenia spectrum disorders or other psychotic disorders and caregivers had to be actively involved and alert to new messages and react within a certain time and period agreed upon. For the caregivers, the benefits of the experience have been to be able to identify worse functioning earlier and react more quickly. Contrary to what they feared, both the number of face-to-face contacts and shorter contacts by email or telephone increased. For the mental health organization, improvements have included more efficient communications, saving time, and more knowledge regarding the care process. For the future, the authors suggest that the tool should be integrated into the daily routine of caregivers, synchronized with the electronic patient record and upgraded with new functionalities like screen-to-screen communications, short message service reminders, electronic dictionary of medical concepts, shared-decision making, and self-management modules for people experiencing schizophrenia spectrum disorders or other psychotic disorders [47].

##### Schizophrenia-Window-of-Hope.Com

This South African Internet resource was dedicated to people experiencing schizophrenia spectrum disorders or other psychotic disorders with the expectation that an Internet resource would be beneficial in terms of psychoeducation and information for people living far away from health centers and for those experiencing stigma. The benefits, analyzed through informal feedback, have been to extend the information and education possibilities beyond the clinical consultation, which has obvious time constraints, and to offer anonymity to users [48].

##### e-Motional Training

An online program addressing the social cognition of people experiencing schizophrenia spectrum disorders or other psychotic disorders in Spain, this neuropsychological rehabilitation based on short duration exercises, tutorials, and a short animated film was shown to be a viable, easy to understand, and pleasant program with significant results on several social cognition variables [41].

##### Horyzon

This Australian moderated online social therapy for long-term recovery in first episode psychosis, designed for computer and Internet-enabled mobile devices (smartphones and tablets) and using peer-to-peer social networking, individually tailored interactive psychosocial interventions, and the involvement of expert and peer moderators, showed a high system usage with no incidents and a significant reduction in depressive symptoms [35].

##### Self-Management and Recovery Technology Therapy

This digital protocol designed to promote recovery in psychosis has been elaborated, following key processes in self-management and recovery as well as symptom monitoring, coping enhancement, and behavior change material, around 7 topics: recovery, managing stress, physical health, “me,” empowerment, relationships, and life. The Self-Management and Recovery Technology (SMART) sessions are delivered through a website accessible via tablets with a mental health worker being present and assisting the self-management of the tool. The intervention is currently being evaluated through a randomized controlled trial [44].

##### Web-Based Informed Consent Process

Even the informed consent process is now tested in Web-based versions as tools for enhancing the informed consent process in schizophrenia research. A Web-based protocol has proved to be not only feasible but more effective than printed consent forms because of greater interactivity and flexibility. The other asset of this kind of process is that it is easily disseminated [49].

### Benefits and Challenges of Internet Use for People Experiencing Schizophrenia Spectrum Disorders or Other Psychotic Disorders

#### Benefits of Internet Use for This Population

##### Empowerment Through Knowledge and Peer-to-Peer Help

Authors generally converge to say that the use of the Internet empowers people experiencing schizophrenia spectrum disorders or other psychotic disorders by allowing them to better understanding their disease and gaining knowledge [32]. Also, robust associations have been identified between peer support, empowerment, and recovery [17]. Online peer-to-peer forums enhance coping, self-esteem, and reassurance [33] and provide relief from information exchange and support [32]. They are likely to produce positive outcomes, especially when professionally moderated and focused on self-efficacy, problem solving, and social recovery [14].

##### Challenging Stigma and Self-Stigma

As a population suffering from especially strong stigma [20-23] and self-stigma [21], a way of internalizing negative stereotypes [50-53], people experiencing schizophrenia spectrum disorders or other psychotic disorders may find ways to challenge those adverse stereotypes thanks to Internet use or Internet interventions. Precisely, interacting with peers online can create greater social connectedness and feelings of group belonging that strengthen stigma resistance [54]. The Internet has the capacity to easily offer contacts with peers within one’s marginalized group as well as disclosure possibilities, both of those being key dimensions of stigma reduction strategies [55].

##### Well-Adapted Media

The Internet provides better social interactions [56] because it reduces social anxiety [32]. Social opportunities offered by the Internet to people experiencing schizophrenia spectrum disorders or other psychotic disorders represent an interesting research field, as the Web offers a semiprotected and less stigmatized social environment. Affected users have shown no difference compared with control group when creating virtual, realistic, or emotional relationships via the Internet. Research exploring Web-browsing habits of 143 individuals experiencing schizophrenia spectrum disorders or other psychotic disorders compared to those of individuals with nonpsychotic disorders and healthy volunteers has established that almost 80% of the group with psychoses wished to create social connections on the Internet [56]. Although this was tempered by the severity of illness, people experiencing schizophrenia spectrum disorders or other psychotic disorders managed to establish contacts leading to real-life relationships, both friendly and romantic, just as the control group did. This population even formed more virtual relationships with less access to the Internet and less Web-browsing time, as if they were using the majority of their time to form social connections. The Internet seems useful for them because there is not the same need for preliminary social skills and motivation. The authors concluded that the Internet represents a promising tool for rehabilitating people experiencing schizophrenia spectrum disorders or other psychotic disorders by providing an accessible platform to social interactions [56].

The other characteristics of the Internet that seem beneficial for people experiencing schizophrenia spectrum disorders or other psychotic disorders are the absence of hierarchy and the limited level of commitment [32]. Also, the Web’s technical specificities facilitate multimodal expression including sound, image, and text in an idiosyncratic way that allows the expression of emotions about the disease that seems to suit people experiencing schizophrenia spectrum disorders or other psychotic disorders [32].

##### More Available Care

As we stressed earlier, the Internet, through e-mental health, offers flexibility and connectivity, which means more available care [57]. This is particularly valuable for those who have difficulties accessing health and mental health care either because of a rural environment or because of limitations due to anticipated discrimination and self-stigma that prevent them from going to the doctor [40,58]. This is also important with regard to specific treatment deliveries, such as cognitive behavior therapy, for which there are a limited number of trained clinicians [40].

##### Efficacy and Clinical Improvement

In general, recent studies highlight the benefits for people experiencing schizophrenia spectrum disorders or other psychotic disorders of using Web-based support systems, for example, by assessing service users’ physical, psychological, and social conditions despite their cognitive problems [59]. Recent experiences show that innovative and flexible interventions that integrate different technologies (eg, mobile phones, chat rooms), evidence-based therapy, and peer and professional support are likely to be the most acceptable and effective for users [14]. Also, the general conclusion of these protocols is that Internet-based support has the potential to foster global recovery in people experiencing schizophrenia spectrum disorders or other psychotic disorders beyond what is possible in traditional interventions without interfering with face-to-face mental health care: it supplements existing services and augments traditional relationships rather than replacing them [14,34,35].

That being said, while acceptability and feasibility of Internet-based interventions designed for people experiencing schizophrenia spectrum disorders or other psychotic disorders have been well established by several recent studies [27,28,34,60-64], reviews in this field of research have concluded that only a few studies have shown clinical efficacy with a robust methodology and on a quantitatively sufficient population [34,39]; also, people included in the studies were generally clinically stable, which could represent a bias in this area. Although very promising in terms of outcomes, Internet-based interventions including online, social media, and mobile technologies demand further investigation through larger controlled studies [34].

#### Challenges of Internet Use for This Population

##### The Disease Itself

Specific difficulties that people experiencing schizophrenia spectrum disorders or other psychotic disorders may encounter with the Internet as a source of information are stimulus overflow, incapacity to overcome the abundance of information, lack of concentration, lack of energy, paranoid ideas, reactive symptoms, and the need to distance themselves from illness-related topics in order to enhance recovery [32]. Also, a study conducted in Greece on 30 outpatients experiencing psychosis and a control group of 71 participants from the general population has underlined that the risk of Internet addiction could be higher for those with a psychosis, a low self-esteem, and difficult interpersonal relationships [65].

With a highly probable link to the disease, there are other reasons not to go on the Internet to seek information, such as lack of access, financial problems, difficulties in the use of technology, fear of viruses, fear of addiction, preference of other sources, expectation of low quality, information request already satisfied through another mean, lack of interest, or preference to receive information from the doctor [32].

##### Perceived Impact on the Relationship With the Doctor

Another difficulty encountered by people experiencing schizophrenia spectrum disorders or other psychotic disorders using the Internet as a source of information is related to their subjective perception that the Internet could modify their attitude toward medication and their relationship to their doctor. Regarding the medication, Internet-retrieved information, especially about side effects, could lead to a more critical attitude toward one’s own medication. Therefore, the information found on the Internet should be discussed with the doctor, but it is far from being systematically the case, mainly because the people experiencing schizophrenia fear that doctors could feel criticized and that they will not change their vision. However, the hierarchic relationship is perceived as modified by the use of the Internet as a source of information by the patient. These relationship changes depend on the quality of the former relationship [32].

##### Flaws in Dedicated Internet Sites

As Schrank and colleagues [32] have stressed, the nature of Internet sites dedicated to schizophrenia and psychoses can be problematic as well. Indeed, difficulties related not to the people experiencing schizophrenia spectrum disorders or other psychotic disorders themselves but to the sites dedicated to schizophrenia are that these are generally difficult to read. Other authors confirm that these sites are especially difficult to read and navigate [66,67]. Also, the available information is often limited. In an investigation of what schizophrenia-related information is available on the Internet, a survey of 21 schizophrenia sites showed that although 67% of them did contain information about related health problems such as weight gain due to medication, in more than a half of these sites, this content was limited to user-generated comments and represented less than one paragraph of information [2].

Also of concern is the relationship between drug company funding of websites and their content; the study by Read [3] highlighted that 58% of schizophrenia websites receive funding from drug companies and that these sites are more likely to espouse biogenetic causes rather than psychosocial ones; emphasize medication rather than psychosocial treatment; portray schizophrenia as a debilitating, devastating, and long-term illness; and link violence to quitting medication. In conclusion, drug company money has an impact on the content of websites in the sense that perspectives and statements expected to increase drug sales are more likely to appear on drug company–funded websites [3].

In general, there is a lack of ethical control, monitoring, and evaluation of mental health websites [68], which makes their use risky for young people experiencing mental illness who are used to navigating online on their own, often for some years [69]. There are not enough security standards [70]; for example, only 50% of Web-based information on the treatment of schizophrenia and attention deficit hyperactivity disorder advises to clarify information with a professional [66]. These risks are important to take into consideration when it comes to vulnerable populations.

Although interactivity is generally sought by people experiencing schizophrenia spectrum disorders or other psychotic disorders, sites devoted to schizophrenia and psychoses offer limited interactivity when compared to other information sites [71].

##### Attrition Rate of e-Mental Health Programs

In the field of other mental diseases such as bipolar disorder, an obstacle constituted by the high rate of attrition of the programs has been identified in the process of e-mental health care and delivery. Indeed, in an attempt to explore the phenomenon of participant adherence, an Australian study surveyed 358 people included in an online psychoeducation program for individuals newly diagnosed with bipolar disorder [72]. The results showed that many of the reasons for leaving the program were independent from program factors (for example, acute phases of bipolar disorder or clinical recruitment setting). But some program specificities had an influence on attrition, in particular the information being too general and not personally tailored or supported by an expert patient “informed supporter.” The attrition rate (26.5%) was equivalent to the one of the other Internet interventions (31% average) from the systematic review of 19 Internet-based psychological treatment programs, but, surprisingly, lower than the rate found in a meta-analysis of 125 studies about psychotherapy programs delivered face-to-face. While these results demand to be verified on a target population of persons experiencing schizophrenia or other psychotic disorders, they appear in favor of personalized and guided interventions rather than fully automated interventions [72].

## Discussion

### Summary

Even if the Internet has shown its effectiveness in many areas, the “Internet paradox” is a new paradigm telling us that it could in the long term lead to a decline in general mental well-being, especially through the use of social networks like Facebook or other networks [11,73]. Developing its access and functionalities for people experiencing schizophrenia spectrum disorders or other psychotic disorders might then seem risky or useless.

However, our review of the literature suggests that despite the ethical and security issues raised by some studies [66,68,70], the Internet is likely to offer mental health services that are effective as interventions [14,37,45,48,59] and relapse prevention tools [10,14,45,47] in the field of severe mental disorders such as schizophrenia spectrum disorders or other psychotic disorders. Getting health information on the Internet and being able to manage one’s mental disorder with Web-based tools helps people experiencing schizophrenia spectrum disorders or other psychotic disorders to participate to medical decision-making processes, gain empowerment, and balance relationships with doctors [9,32,33]. In addition, the Internet might well propose specific opportunities in terms of social interactions for people experiencing those illnesses, who are often impaired in the social domain [14,56].

Yet, according to our literature review, information about the way people experiencing schizophrenia spectrum disorders or other psychotic disorders use the Internet regarding their health is not available in every country. As we mentioned in our introduction, the proportion of Internet users in this target population is higher in the most recent studies, which seems to reflect the evolution in the general population. The rates given by the available studies as well as rates of mental health information–seeking activities among Internet users experiencing those conditions, when possible, are presented in Table 1.

This should lead us to ask ourselves if similar proportions are to be observed in other countries with similar Internet access possibilities. This area of research requires further development in order to ascertain if, depending on the country they live in, the habits of people experiencing schizophrenia spectrum disorders or other psychotic disorders vary in their use of the Internet, if they are able to find the content that they are looking for, and if the specific developments and tools to be used are plenty or scarce. Of course, the fact that no study has been published on the matter in a given country does not mean that there is no available Internet-based content that persons experiencing schizophrenia spectrum disorders or other psychotic disorders can access regularly to find information about their mental health or even e-mental health support.

**Table 1:** Internet use of people experiencing schizophrenia spectrum disorders or other psychotic disorders by country of research.

Service users	Switzerland [25] N=319 n (%)	United States [24] N=100 n (%)	United States [27] N=80 n (%)	Australia, New Zealand [1] N=71 n (%)	Germany [26] N=337 n (%)
Percentage using the Web	200 (62.6)	34 (34)	80 (48)	—	268 (79.5)
Percentage seeking health information online	137 (42.9)	19 (53)	—	71 (35.2)	190 (56.4)

However, a country-by-country approach remains limited in its conception, as the Internet is by definition a worldwide platform as long as one has a sufficient knowledge of English. From our sources, papers focusing on ethical and security issues in the use of the Internet by people experiencing psychiatric conditions like schizophrenia spectrum disorders or other psychotic disorders had all adopted a global approach and had found similar challenges in the studied dedicated Internet sites without establishing any specificity according to their geographic origin [68,70,74]. Therefore, the Internet’s nature itself could explain that research studies analyzing advantages and challenges for people experiencing schizophrenia spectrum disorders or other psychotic disorders using the Internet about their mental health prefer to address issues globally. Yet, specificities in the access to the Internet and new technologies linked to the habits and preferences of this target population remains interesting to evaluate with a country-specific approach from a public health perspective.

Future research studies could also usefully aim to confirm if there is a link between diagnosis and use of the Internet. Indeed, this link remains insufficiently explored, namely by a few studies that found contradictory results. For example, an Australian survey conducted in 2011 on 71 people experiencing schizophrenia spectrum disorders or other psychotic disorders recruited from both community and inpatient settings and 238 general practice attendees on their use of different media to obtain information on health matters found that people with psychotic disorders had less access to the Internet due to financial difficulties, a frequently itinerant way of life, and a lower educational level. Only those with higher education placed an important level of trust in the Internet as a health information source [1].

Another study conducted in Germany involving 337 people recruited in a psychiatric facility showed on the contrary that the Internet use of the psychiatric population corresponded to the use of the general population and that there was no significant difference between the included diagnoses, that is to say schizophrenia and affective, neurotic, stress-related, and somatoform disorders [26,30]. The different methodologies and populations used in these research studies do not allow direct comparisons but imply that there is a need to further explore these results.

The limits of our review are related to the diversity of populations used in the articles reviewed, with different types of recruitments (within psychiatric facilities or not) and sometimes multiple terminologies or even diagnosis being considered. Indeed, we have chosen to include pertinent or important studies that sometimes used other terminologies than our defined focus (schizophrenia spectrum disorders or other psychotic disorders), for example, “psychosis,” “severe or serious mental illness,” or “serious psychiatric conditions or disorders.” While such terminologies include schizophrenia spectrum disorders and other psychotic disorders, some of these studies (although rare in our review) focused on broader populations, for example, people experiencing bipolar disorders with psychotic features, which could represent a bias. Another difficulty is the fact that most of the research studies discussed in our review concern small to very small populations. Also, the small number of studies focusing on specific countries and their offerings in this area could not allow us to establish reliable comparisons between them. Furthermore, being a literature review, our study lacks an up-to-date clinical perspective that would aim to investigate some of the research leads identified in this section. Finally, an important limit to our research is that in the field of new technologies things evolve quickly and have to be constantly reevaluated.

### Conclusion and Recommendations

Many issues are raised concerning the access to the Internet by people experiencing schizophrenia spectrum disorders or other psychotic disorders, as well as the impact of the Internet as a source of mental health information; the compliance and relationship with doctors; and the readability, quality, and security of specialized websites and interventions. However, the benefits seem to outbalance the disadvantages as far as users’ wishes and needs are addressed. In any case, the available literature has established that the Internet is already an important and influential source of information and support for people experiencing schizophrenia spectrum disorders or other psychotic disorders and that a significant proportion of users have shown an interest for Internet interventions in the context of their treatment. Most of the existing interventions have shown good feasibility and acceptability. The remaining challenge is to strengthen and generalize the evaluation regarding clinical efficacy. Taking that evolution into account, we have outlined in this paper some recommendations that seem key to us to continue the move toward e-mental health, which alongside with the traditional system will shape the future of care for persons experiencing schizophrenia spectrum disorders or other psychotic disorders:

1. Generalize structured and moderated access to the Internet for people experiencing schizophrenia spectrum disorders or other psychotic disorders.
2. Inform doctors about alternative sources of information to suggest (or not) to people experiencing schizophrenia spectrum disorders or other psychotic disorders. Some authors suggest that doctors should be knowledgeable not only about medication and secondary effects but also about alternative sources of information available for people experiencing schizophrenia spectrum disorders or other psychotic disorders on this matter, and they should in particular be able to advise service users about what is or is not available on the Internet and to advise the creators of schizophrenia websites about what kind of information is needed.
3. Involve doctors and integrate the insights from people experiencing schizophrenia spectrum disorders or other psychotic disorders in the creation of specialized websites and in the development of mobile and Web-based applications [39,64].
4. Generalize and extend Web-based interventions integrating different technologies (eg, smartphones, chat rooms with professionals) and evidence-based therapy for a new generation of people experiencing schizophrenia spectrum disorders or other psychotic disorders seeking flexibility and connectivity.
5. Implement a regular structured evaluation of websites and Internet interventions within the scope of national health care authorities in order to ensure quality and prevent harm for people experiencing schizophrenia spectrum disorders or other psychotic disorders by, among other things, extending the use of emergency guidelines and contact information.
6. Tailor and guide Internet-based and all e-mental health interventions to reduce attrition.
7. Invent new types of interventions rather than use online technologies as alternative means to deliver traditional interventions [15].

## Abbreviations

SMART: Self-Management and Recovery Technology
